# Utilizing bioinformatics to identify biomarkers and analyze their expression in relation to immune cell ratios in femoral head necrosis

**DOI:** 10.3389/fphys.2025.1373721

**Published:** 2025-04-16

**Authors:** Dongchen Li, Zhilong Huang, Teng Ma, Yu Su, Zhao Li, Liang Sun, Ming Li, Zhong Li, Yao Li, Qian Wang, Yao Lu

**Affiliations:** ^1^ Honghui Hospital, Xi’an Jiaotong University, Xi’an, China; ^2^ School of Medicine, Yan’an University, Yan’an, China; ^3^ Department of Orthopaedic Surgery, The Nuclear Industry 417 Hospital, Xi’an, China

**Keywords:** necrosis of the femoral head, immune cells infiltration, death-associated genes, biomarkers, bioinformatics

## Abstract

**Background:**

Necrosis of the Femoral Head (NFH) represents a challenging orthopedic condition, characterized by elusive early detection and rapid progression, predominantly in the middle-aged demographic. Current research on the pathophysiological and immunoregulatory mechanisms underpinning immune cell infiltration in NFH is sparse. This study employs bioinformatics analysis of publicly available RNA sequencing databases to elucidate the pivotal molecules and pathways implicated in NFH progression.

**Methods:**

The NFH-related dataset GSE123568 was obtained from the Gene Expression Omnibus (GEO). Subsequently, CIBERSORT was utilized to assess the proportion and distribution of immune cell types, followed by the identification of critical Hub immune cells using LASSO and RFE algorithms. The dataset GSE123568 was then explored to identify significantly differentially expressed genes (DEGs). These genes were further refined by intersecting with death-associated genes reported in existing literature. GO and KEGG pathway enrichment analyses were conducted to elucidate their underlying molecular mechanism. A protein-protein interaction (PPI) network was constructed using the STRING database and visualized via Cytoscape. Hub genes were identified using the CytoHubba plugin, followed by enrichment analysis, and their expression levels were evaluated using the ROC curve. In addition, we performed expression data visualization and ROC curve analysis on the external dataset GSE74089 to further evaluate the discriminative power of the hub genes. Moreover, the study analyzed the correlation between the identified hub genes and Hub immune cells. Finally, we verified the hub genes utilizing real-time fluorescence quantitative polymerase chain reaction (RT-qPCR) and immunohistochemistry.

**Results:**

Four types of immune cells (Neutrophil, Mast cell resting, Myeloid dendritic cell activated, Macrophage M0) were identified. Fourteen pivotal genes (BCL2L1, BIRC2, NFKBIA, XIAP, CFLAR, AKT1, BIRC3, IKBKB, RIPK1, CASP8, TNFRSF1A, IL1B, CASP1, STAT3) were identified, and the findings were validated using the external dataset GSE74089. Among these, STAT3 exhibited the most pronounced positive correlation with neutrophils (r = 0.6804, p = 3.525e-05). Conversely, XIAP displayed the most significant negative correlation with Myeloid dendritic cell activated (r = −0.3610, p = 0.04003). In experiments, the experimental outcomes for five hub genes (CASP8, TNFRSF1A, AKT1, XIAP and STAT3) were congruent with the results obtained from bioinformatics analysis.

**Conclusion:**

Our study identified CASP8, TNFRSF1A, AKT1, XIAP, STAT3 and BCL2L1 as potential biomarkers for NFH patients and elucidated the immune cell types with the strongest association to these markers. These insights may be crucial for the early diagnosis, understanding of the pathophysiological mechanisms, and the development of treatment strategies for NFH.

## Introduction

Necrosis of the Femoral Head (NFH) arises from a multitude of etiologies leading to the disruption or damage of blood circulation in the femoral head (Chen et al., 2022). This results in varying degrees of necrosis in bone cells, bone marrow hematopoietic cells, adipocytes, and other vital components of the femoral head, subsequently inducing alterations in its structure, and manifesting as hip pain, dysfunction, and other symptoms (Tan et al., 2012). Once femoral head collapse occurs in a patient, the progression of the disease becomes challenging to reverse, ultimately evolving into severe hip arthritis within a few years, necessitating total hip arthroplasty (Crim et al., 2021; George and Lane, 2022). The damage to joints and associated tissues in Necrosis of the Femoral Head (NFH) typically precedes the manifestation of clinical symptoms (Larson et al., 2018). While Magnetic Resonance Imaging (MRI) is capable of early NFH diagnosis, the majority of patients are diagnosed in the intermediate or advanced stages of the disease, owing to challenges in early detection of NFH and its rapid progression (Liu et al., 2017; Yuan et al., 2022). Consequently, predicting and preemptively preventing osteonecrosis in high-risk groups for Necrosis of the Femoral Head (NFH) is of paramount importance.

Insufficient studies focused on correlation between NFH and immune inflammation. The bone marrow produces a spectrum of immune cells and factors. Meanwhile immune cells, in turn, regulate bone formation and resorption by secreting relevant cytokines, maintaining the dynamic equilibrium of bone mass (Tsukasaki and Takayanagi, 2019). This denotes a bidirectional regulatory relationship between the two systems (Cai et al., 2022). Notably, the previous research has identified anomalous immune cell infiltration in the peripheral blood samples of patients with NFH (Ma et al., 2019). Furthermore, investigations have substantiated that in the initial stages of Necrosis of the Femoral Head (NFH), osteocytes and osteoblasts are more susceptible to apoptosis than to mere cell necrosis (Calder et al., 2004). Consequently, this study aims to analyze the distribution of immune cell molecules in NFH using high-throughput expression profiling technology. This analysis, in conjunction with examining gene expression patterns associated with cell death, seeks to elucidate the immune mechanisms underlying NFH and identify novel therapeutic targets.

## Materials and methods

### Data search and acquisition

**FIGURE 1:**
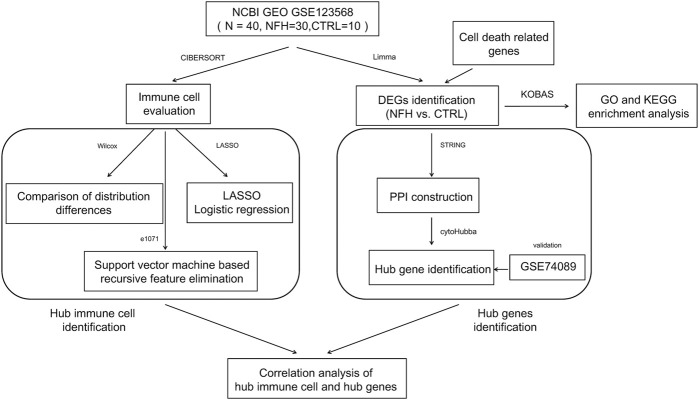
The flow chart of the study.

NFH-related datasets with accession GSE123568, GSE74089 were procured from the NCBI GEO database (Edgar et al., 2002). The GSE123568 dataset comprises samples from 30 NFH patients and 10 healthy controls. Meanwhile, the GSE74089 dataset encompassed cartilage samples from 4 NFH patients and four normal subjects. The flowchart of this study is illustrated in Figure 1.

### Evaluation of Immune Cell Proportions in samples

Immune infiltration denotes the movement and functional integration of immune cells from blood into tissues. Evaluating the proportion of immune cells in affected regions offers potential indicators for subsequent diagnostic applications. Recent research indicates the presence of an aberrant immune infiltrating cell microenvironment in NFH. CIBERSORT(Chen et al., 2018) is a methodology utilized to assess the composition of complex tissue cells via gene expression profiling. In this study, CIBERSORT was employed to predict and assess the proportions of various immune cell types in samples from the GSE123568 dataset. For each sample, the cumulative score of the evaluated immune cell types was equivalent to 1.

### Identification of hub immune cells

The GSE123568 dataset served as the training set in this study, and least absolute shrinkage and selection operator (LASSO) and recursive feature elimination (RFE) was used to screen the immune cell features. The procedural methodology was as follows: Initially, the lars package (v1.2) (Goeman, 2010) was instrumental in conducting regression analysis to identify specific combinations of immune cells. Subsequently, the RFE algorithm within the caret package (Deist et al., 2018) was utilized for further screening of these immune cell combinations. Ultimately, the immune cells identified by both algorithms were cross-referenced with those exhibiting significant distributional differences as determined by the Wilcoxon test, with the intersecting subset designated as the hub immune cells.

### Identification of Significantly Differentially Expressed Genes

The limma package (v3.56.2) (Ritchie et al., 2015) was then employed to identify genes exhibiting significant differential expression between NFH and CTRL, using FDR<0.05 as the selection threshold. Subsequently, the pheatmap package (v1.0.8) (Wang et al., 2014) was utilized for bidirectional hierarchical clustering of the expression values, visualized through a heatmap.

Cell death-related genes were compiled from extant literature (Nan et al., 2022) encompassing autophagy, apoptosis, and pyroptosis (Supplementary Table S1). The significantly DEGs identified were then compared with the 424 cell death-associated genes collated, retaining those that overlapped as significantly DEGs related to cell death. The KEGG Orthology Based Annotation System 3.0 (KOBAS 3.0) database (Bu et al., 2021) was used to conduct GO function and KEGG signaling pathway enrichment analysis on these genes, selecting FDR < 0.05 as the screening threshold.

### Construction of protein-protein interaction (PPI) network

The STRING database (version 11.0) (Szklarczyk et al., 2017) was employed to identify interactions among the protein products of the significantly differentially expressed genes related to cell death, as determined in the previous step. The resultant interaction network was subsequently visualized using Cytoscape (version 3.9.0) (Shannon et al., 2003).

### Identification of hub genes

The cytoHubba plugin (version 0.1) (Chin et al., 2014) in Cytoscape 3.9.0 was utilized for the identification of hub genes within the constructed network. This was achieved using four topological analysis algorithms: MCC (Matthews Correlation Coefficient metric), MNC (Maximal Neighbourhood Coefficient), Degree, and EPC (Edge Percolated Component). The intersecting subset across all four algorithms was designated as Hub genes.

### Efficacy evaluation of hub genes

The expression profile of the hub genes were extracted from the GSE123568 dataset to calculate the distribution of expression between the NFH and CTRL. The ROC (Receiver Operating Characteristic) curve method, utilizing the pROC package (version 1.12.1) (Robin et al., 2011)was employed to evaluate the discriminative capacity of the hub genes in distinguishing samples. Concurrently, the expression data visualization and ROC curve analysis were also conducted on the validation dataset GSE74089 to further assess the discriminative efficacy of the hub genes.

### Correlation analysis between hub immune cells and hub genes

The correlation between hub immune cells and hub genes was computed using the ‘cor’ function.

### Collection of clinical samples

This research complied with the Declaration of Helsinki (revised 2013) (World Medical, 2013) and received approval from the Ethics Committee of the Honghui Hospital affiliated to Xi’an Jiaotong University School of Medicine (No. 202310001). All relevant patients signed informed consent. The study included patients hospitalized between March and December 2023 who met the inclusion criteria. Inclusion criteria: Hospitalized patients diagnosed with NFH, in accordance with the “Clinical Diagnosis and Treatment Guidelines for Avascular Necrosis of the Femoral Head in Chinese Adults (Association Related To Circulation Osseous Chinese Microcirculation Society, 2022).” A total of 5 patients with NFH and 5 control subjects were included in the study. The inclusion criteria were as follows: (1) diagnosed with ARCO stage III or IV NFH, presenting with severe hip joint pain and accompanying limping; (2) willingness to undergo total hip arthroplasty; (3) age ≥18 years; (4) generally healthy with no severe hereditary familial diseases and normal results in physical examination. Exclusion criteria included: (1) non-conformance with the above diagnoses, staging, and inclusion criteria; (2) malignancies; (3) hip joint infections; (4) inability to provide sufficient protein from femoral head samples for testing due to various reasons. The control group consisted of patients who underwent total hip arthroplasty within 24 h post-trauma due to femoral neck fractures.

### Real-time quantitative polymerase chain reaction assay validation

A total of 10 whole blood samples were collected (including 35 NFH samples and 5 from a normal control group).

Blood samples were collected and processed according to the relevant protocol for peripheral blood mononuclear cell (PBMC) extraction (Saadatian et al., 2023), which were then lysed in Trizol (AC0101, SparkJade, China). Total RNA from PBMCs was isolated using the RNAsimple Total RNA Kit (DP419, TianGen, China). The RNA quality and concentration were determined using NanoDrop (Thermo Fisher Scientific, US). Reverse transcription was performed using 1 µg of total RNA with the Reverse Transcription Kit (K1622, Thermo Fisher Scientific, US) to obtain cDNA. Finally, quantitative analysis was conducted using the CFX96 Real-Time PCR Detection System (CFX96, BIO-RAD, USA) through qRT-PCR.

Amplification conditions were as follows: initial denaturation at 95°C for 5 min, followed by 40 cycles of 95°C for 10 s, 60°C for 20 s, and 72°C for 20 s. Subsequently, a melting curve was established to obtain the final results. GAPDH was used as the reference gene, and the 2^−ΔΔCt^ method was employed to calculate the relative expression levels of the target genes. The primers used in this study were synthesized by Beijing Qingke Biological Technology Co., Ltd.

### Immunohistochemistry

**FIGURE 2:**
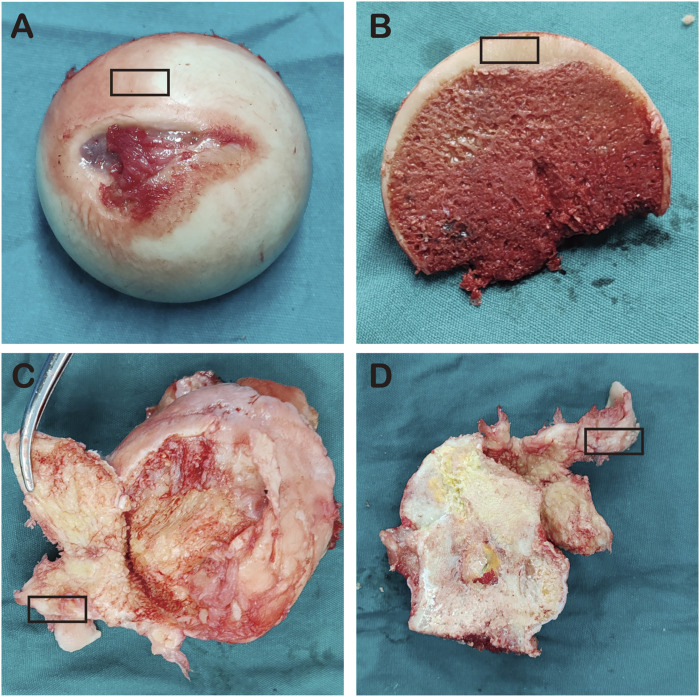
**(A–B)** represent the femoral heads of NFH patients; **(C–D)** represent the femoral heads of the control group, and the black box indicates cartilage area collected from the femoral heads.

Articular cartilage tissue was obtained from the anterior superior part of the femoral head of patients in both groups (5 NFH group, 5 control group), particularly from the cartilage collapse area, as shown in Figure 2.

Cartilage tissue samples fixed in paraformaldehyde were decalcified and embedded in paraffin. Subsequently, the paraffin-embedded cartilage tissues were sectioned into 5 μm thick slices and placed on glass slides. The initial process involved deparaffinizing the cartilage tissues on the slides in xylene, followed by antigen retrieval and graded ethanol hydration. The sections were then treated with a 3% hydrogen peroxide solution, followed by serum blocking, and subsequently incubated overnight at 4°C with antibodies against STAT3 (ET1607-38), XIAP (ER65320), TNFRSF1A (HA500140), CASP8 (ET1603-16), and AKT1 (ET1609-47) at a dilution of 1:50 (HuaBio, China). After washing with PBS, the sections were incubated with the corresponding secondary antibodies and stained with 3,3′-diaminobenzidine (DAB), followed by counterstaining with hematoxylin. The percentage of positive chondrocytes in the sections was assessed, and the t-test was employed to evaluate significant differences in the expression of STAT3, XIAP, TNFRSF1A, CASP8, and AKT1 proteins between NFH patients and controls.

### Statistical analysis

All data processing and statistical analysis were performed using R3.6.1 software. Wilcoxon rank sum test was used to compare the differences in the distribution of different immune cells between NFH and CTRL. Pearson correlation analysis was used to determine the correlation between differentially expressed cell death-related genes and immune infiltration. The biomarkers expression levels in qRT-PCR and immunohistochemistry results were analyzed using the independent sample Student's t-test.

## Results

### Evaluation of Immune Cell Proportions in sample

**FIGURE 3:**
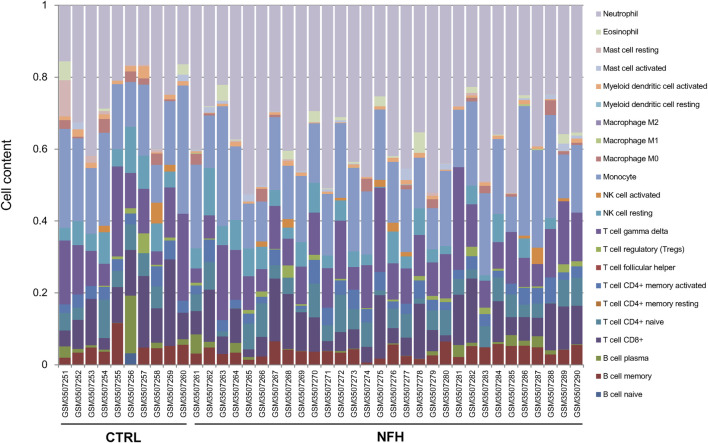
Distribution of Immune Cell Proportions in the Sample. Each type of immune cell is represented by a distinct color.

**FIGURE 4:**
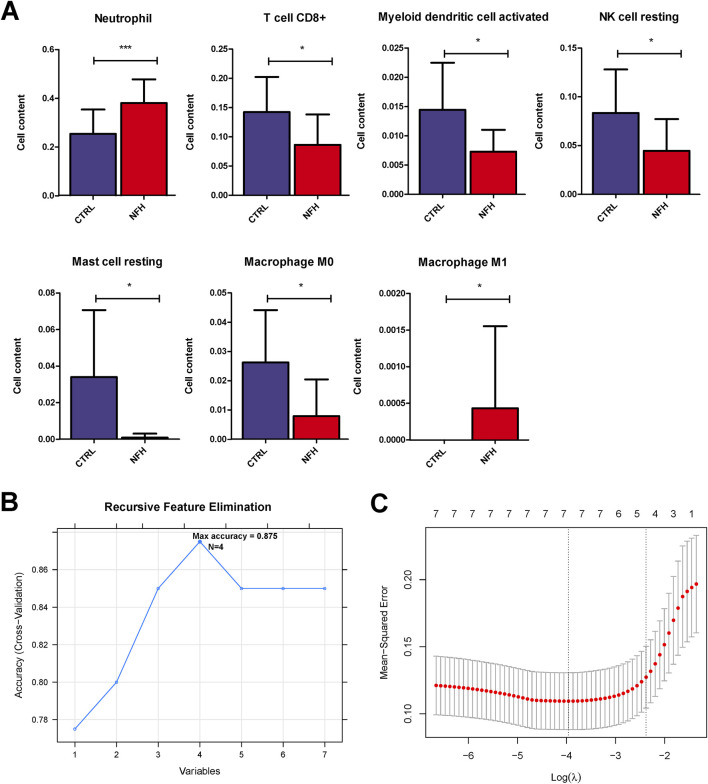
**(A)** horizontal distribution Histograms of seven immune cell types with significant distributional Differences. *:p < 0.05; **:p < 0.01; ***:p < 0.005. **(B)** immune cells screened by the SVM-RFE Algorithm. **(C)** selection of immune cells using the lasso model.

Using the gene expression profile data from the GSE12368 samples (Supplementary Table S2), CIBERSORT was employed to determine the distribution of immune cell types in each sample, leading to the identification of 22 immune cell types (Supplementary Table S3). The immune cell composition of each sample was depicted in Figure 3. A total of 7 significant different immune cells were discerned: Neutrophil, T cell CD8^+^, Myeloid dendritic cell activated, NK cell resting, Mast cell resting, Macrophage M0, and Macrophage M1. Compared to the control group, the NFH samples showed increased levels of Neutrophils and Macrophage M1, while the levels of T cell CD8^+^, Myeloid dendritic cell activated, NK cell resting, Mast cell resting, and Macrophage M0 were reduced (Figure 4A).

### Identification of hub immune cells

By utilizing the LASSO and RFE algorithms, four immune cell types were identified: Neutrophil, Mast cell resting, Myeloid dendritic cell activated, and Macrophage M0 (Supplementary Table S4). The specific parameters for each algorithm are elaborated in Figure 4B.

### Screening of significantly differentially expressed genes

**FIGURE 5:**
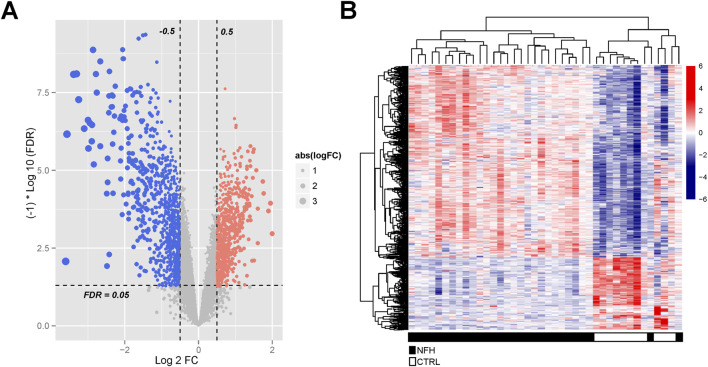
**(A)** Identification of Significantly Differentially Expressed Genes in the GSE123568 Dataset of NFH. The volcano plot displays all DEGs, with blue and red dots signifying significantly downregulated and upregulated genes, respectively. The black horizontal line indicates an FDR of 0.05.; **(B)** Heatmap Illustrating the DEGs Between the Two Groups.

**FIGURE 6:**
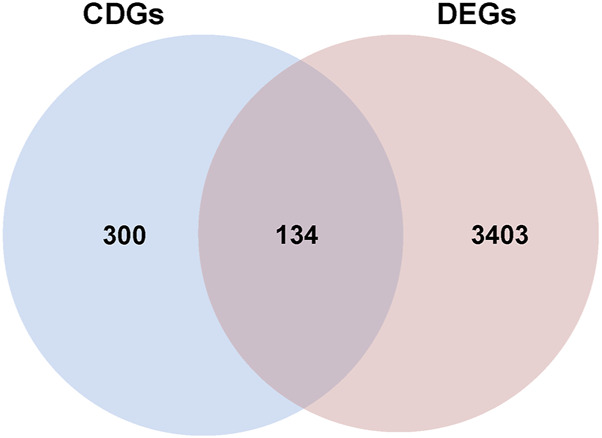
The Venn diagram of overlapping differential genes between CDGs and DEGs.

A total of 3537 differentially expressed genes (DEGs), including 956 downregulated and 2581 upregulated genes, were identified using the limma package (Supplementary Table S5), as demonstrated in the volcano plot (Figure 5A). A heat map illustrating sample clustering based on these DEGs was subsequently created (Figure 4B). Comparison of these DEGs with 434 cell death-related genes (CDGs) from the literature revealed 134 intersecting genes (Supplementary Table S6), shown in Figure 6.

**FIGURE 7:**
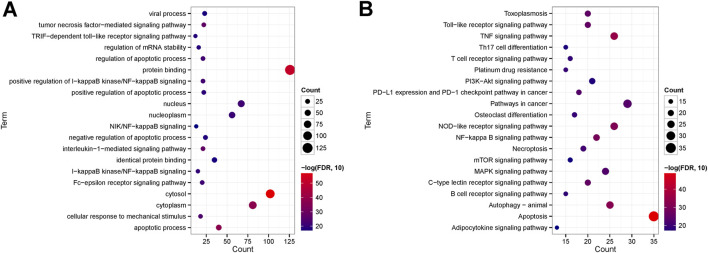
**(A)** Top 20 GO enriched terms; **(B)** Top 20 KEGG enriched pathways.

Further investigations entailed GO function and KEGG signaling pathway enrichment analyses on the intersecting DEGs. These analyses uncovered 411 significantly enriched GO function nodes and 127 KEGG signaling pathways, highlighting the top 20 enriched function nodes and pathways (Supplementary Table S7). The GO enrichment analysis underscored significant involvement in biological processes like the apoptotic process, interleukin-1-mediated signaling pathway, tumor necrosis factor-mediated signaling pathway, regulation of the apoptotic process, and mRNA stability regulation. Also, cell components such as the cytosol, cytoplasm, nucleus, and nucleoplasm were prominently enriched. Molecular functions including protein binding and identical protein binding were notably enriched (Figure 7A). KEGG pathway analysis indicated enrichment in pathways like Apoptosis, Necroptosis, TNF signaling pathway, NF-kappa B signaling pathway, T cell receptor signaling pathway, B cell receptor signaling pathway, PI3K-Akt signaling pathway, Th17 cell differentiation, and Adipocytokine signaling pathway (Figure 7B).

### Construction of PPI network

**FIGURE 8:**
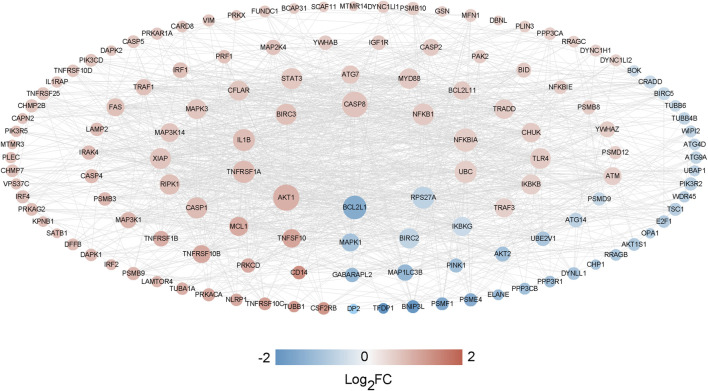
The PPI network analysis between CDGs and DEGs.

A thorough analysis of 134 significantly differentially expressed genes related to cell death (with a confidence threshold of 0.400) was performed to outline potential protein-protein interactions using the STRING online database. By retaining interaction connections with a score above 0.4, a total of 1262 interaction pairs were identified (Supplementary Table S8), leading to the formation of the interaction network (Figure 8).

### Identification of hub genes

**FIGURE 9:**
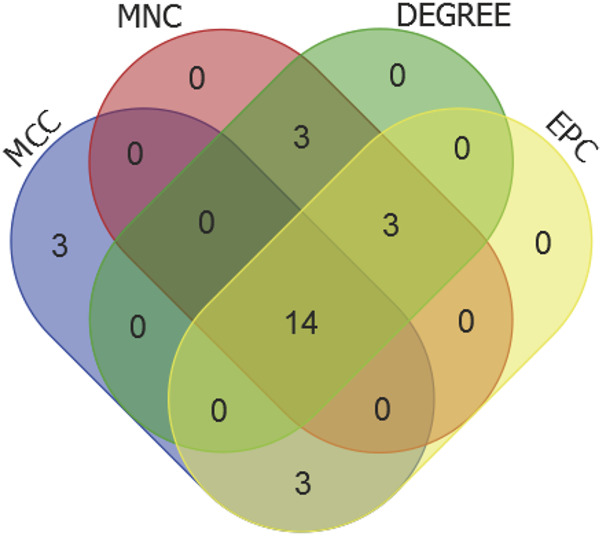
Venn diagram of the top20 candidate hub genes of MCC, MNC, DEGREE and EPC algorithms.

**FIGURE 10:**
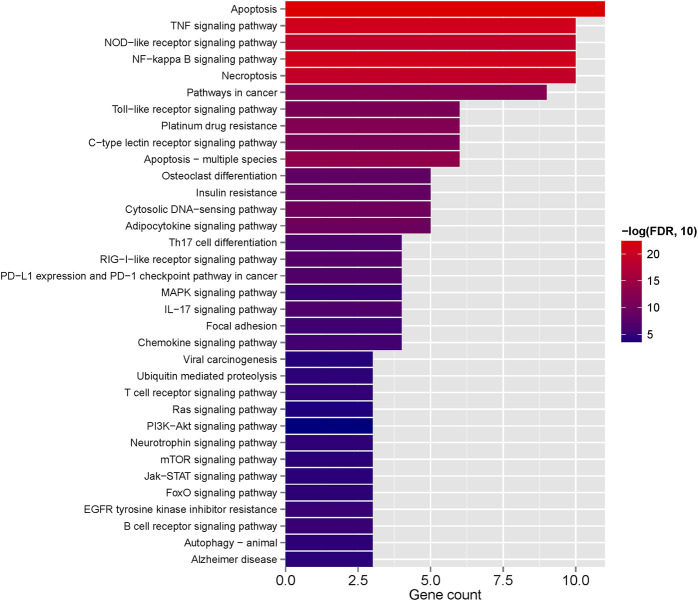
The KEGG enriched pathway of 14 hub genes.

We selected the top 20 genes from each algorithm as potential hub genes (Supplementary Table S9). Fourteen intersecting genes were then identified: BCL2L1, BIRC2, NFKBIA, XIAP, CFLAR, AKT1, BIRC3, IKBKB, RIPK1, CASP8, TNFRSF1A, IL1B, CASP1, STAT3 (Figure 9). Further analysis through KEGG signaling pathway enrichment showed that these 14 hub genes were predominantly linked to apoptosis, the TNF signaling pathway, and necroptosis (Figure 10).

### Assessment of hub gene expression levels

**FIGURE 11:**
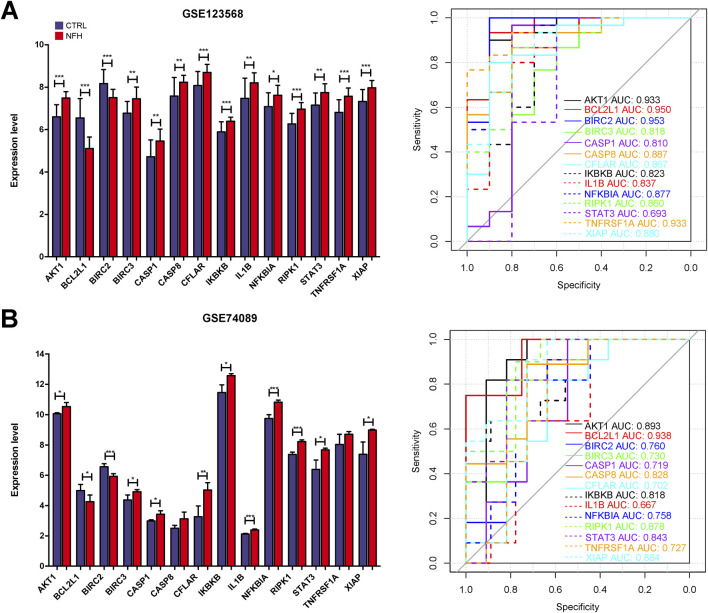
**(A)** The expression levels of 14 Hub genes in GSE123568 were depicted, and their diagnostic validity was corroborated through the ROC curve analysis. **(B)** The expression levels of 14 Hub genes in GSE74089 were depicted, and their diagnostic validity was corroborated through the ROC curve analysis. *:p < 0.05, **:p < 0.01, ***:p < 0.005.

The expression profiles of 14 hub genes, as illustrated in Figure 11, were derived from the dataset. Validation results using an external dataset (GSE74089) revealed significant differences in the expression levels of these 14 hub genes, consistent with the patterns noted in the training dataset GSE123568 (Supplementary Table S11). ROC curves were then generated for both the training dataset GSE123568 and the validation dataset GSE74089 to evaluate the diagnostic accuracy of these hub genes. The AUC values for these genes were above 0.6, confirming their superior predictive power. This evidence supports the potential of these hub genes as effective biomarkers for the diagnosis of NFH (Figure 10).

### Correlation analysis between hub immune cells and hub genes

**FIGURE 12:**
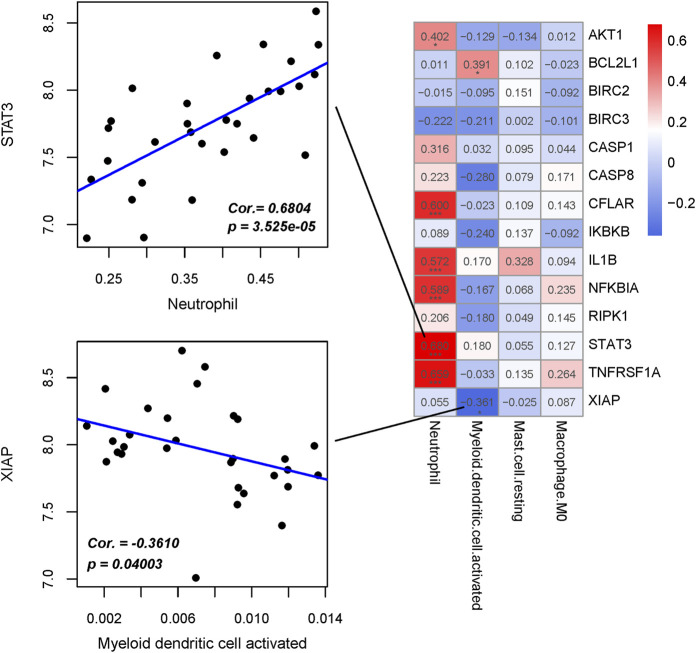
Interrelationship between Hub immune cells, Hub genes and involved KEGG signaling pathways.

**FIGURE 13:**
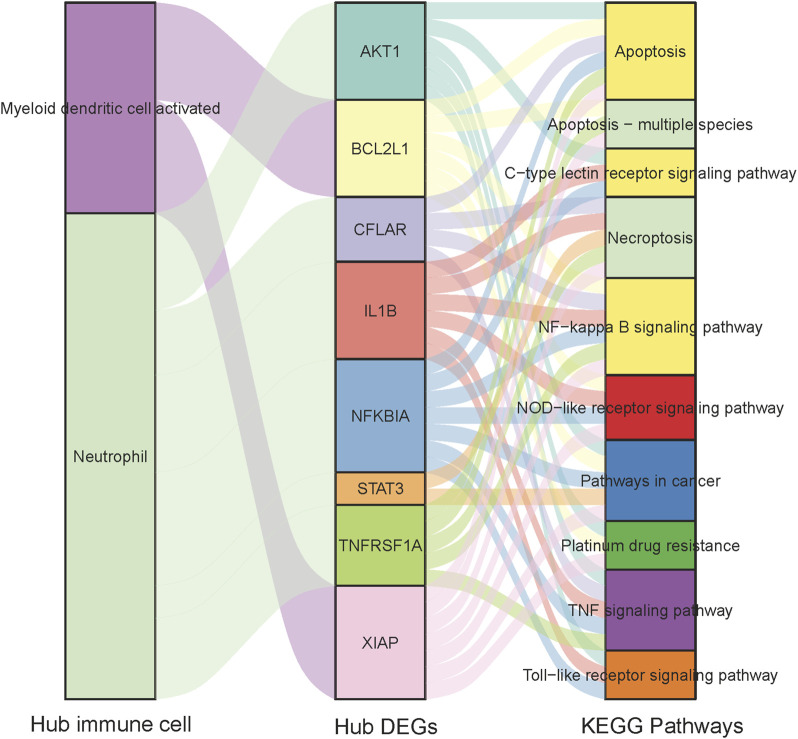
Heat map of the correlation between Hub immune cells and Hub genes.

The correlation between four hub immune cells and 14 hub genes was determined (Figure 12). The analysis revealed that STAT3 exhibited the strongest positive correlation with neutrophil cells (r = 0.6804, p = 3.525e-05), while XIAP demonstrated the most pronounced negative correlation with activated myeloid dendritic cells (r = −0.3610, p = 0.04003) (Supplementary Table S12). Additionally, the interplay among immune cells, hub genes, and their associated KEGG-enriched signaling pathways was elucidated using a Sankey diagram (Figure 13).

### Real-time quantitative polymerase chain reaction

**FIGURE 14:**
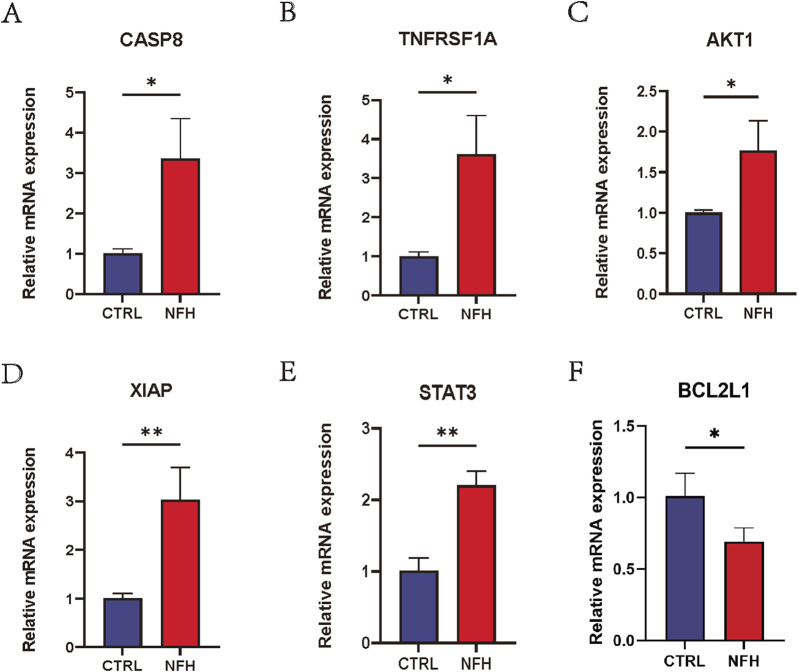
**(A–F)** Relative mRNA expression levels of CASP8, TNFRSF1A, AKT1, XIAP, STAT3 and BCL2L1 in PBMCs of NFH patients and healthy individuals. *: p < 0.05, **: p < 0.01, ***: p < 0.005.

The study results indicate that in the NFH group, the expression levels of CASP8, TNFRSF1A, AKT1, XIAP, STAT3 and BCL2L1 were significantly higher than those in the control group. This observation is consistent with previous bioinformatics analysis, confirming the reproducibility and reliability of these six hub genes. Detailed results are presented in Figure 14.

### Immunohistochemistry

**FIGURE 15:**
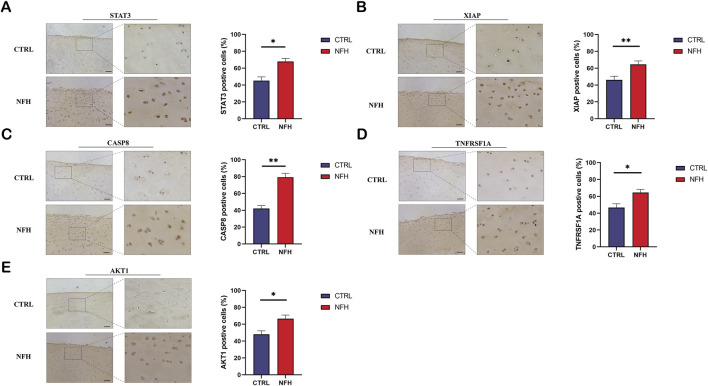
**(A–E)** respectively demonstrate the immunohistochemical results for STAT3, XIAP, TNFRSF1A, CASP8, and AKT1 proteins in the cartilage of NFH patients compared to normal cartilage in the control group. Scale bar = 200 μm.

The expression of STAT3, XIAP, TNFRSF1A, CASP8, and AKT1 proteins in NFH and normal cartilage was assessed through immunohistochemistry analyses, as shown in Figure 15. The trends observed were consistent with those of the qPCR results. The expression levels of these five proteins were significantly higher in NFH than in the control group, a finding that aligns with bioinformatics analysis results.

## Discussion

NFH is a progressive condition characterized by an inadequate blood supply to the necrotic region, culminating in osteocyte mortality. This sequence of events precipitates the progressive collapse of the femoral head and subsequent joint destruction, ultimately progressing to secondary hip arthritis (Cui et al., 2021; Motta et al., 2022). Inflammation and osteocyte death are the key pathogenic factors of NFH. (Konarski et al., 2022); however, the precise molecular mechanisms underlying their contribution to NFH remain obscure. Post-onset of NFH, necrotic tissue discharges copious cytokines and chemokines, orchestrating the infiltration of inflammatory cells into the affected region, disrupting the existing immune equilibrium, and culminating in extensive osteocyte demise, which leads to the collapse of the femoral head. Consequently, the pathogenesis of NFH is intimately associated with immune infiltration (Ma et al., 2019; Wang et al., 2021). M1 macrophages have been demonstrated to be closely associated with the progression of NFH(Cheng et al., 2023).

In this study, a total of 134 DEGs were obtained based on the intersection of differentially expressed genes in the GSE123568 data set and CDGs in the published literature. GO results showed that DEGs were significantly enriched in apoptosis, necroptosis, and inflammation-related pathways (e.g., interleukin-1-mediated signaling pathway, tumor necrosis factor-mediated signaling pathway). Previous studies have shown that the initial death in the tissue from patients with osteonecrosis, is the apoptosis of osteoblasts and osteocytes, rather than their simple necrosis of osteoblasts and osteocytes (Calder et al., 2004; Plotkin et al., 2007). KEGG enrichment results showed that these genes were mainly related to Apoptosis, Necroptosis, TNF signaling pathway, NOD-like receptor signaling pathway, PI3K-Akt signaling pathway and et al. NOD-like receptor signaling pathway is closely related to immune response. Research has substantiated that M1 macrophages demonstrate a significant correlation with the progression of NFH (Cheng et al., 2023). Furthermore, it has been observed that in macrophages, aberrant activation of the TLR4/NLRP3/GSDMD signaling pathway induces pyroptosis, resulting in atypical accumulation of inflammatory cells in the affected region, thereby markedly exacerbating the inflammatory response (Xu et al., 2021).

We next, constructed the PPI network of differentially expressed genes involved in NFH by STRING database, and 14 hub genes including BCL2L1, BIRC2, NFKBIA, XIAP, CFLAR, AKT1, BIRC3, IKBKB, RIPK1, CASP8, TNFRSF1A, IL1B, CASP1 and STAT3 were identified. KEGG enrichment analysis was used to discuss the molecular mechanism of these 14 hub genes. Reveals these genes were mainly enriched in Apoptosis, TNF signaling pathway, NOD-like receptor signaling pathway, F-kappa B signaling pathway and necrotosis. The enriched pathways basically revolve around ' apoptosis ' and ' necroptosis '. Related studies have found that the level of tumor necrosis factor-α (TNF-α) in the serum of patients with NFH is higher than that of normal people, and the high level of TNF-α promotes the apoptosis of osteoblasts (Zheng et al., 2020). The activation and propagation of the NF-kappa B signaling pathway are pivotal in the formation and activation of osteoclasts (Wu et al., 2019). Excessive activation of osteoclasts can compromise the structural integrity and strength of the femoral head, potentially resulting in its collapse and necrosis (Chen et al., 2020). The expression levels of these 14 Hub genes were identified from the GSE123568 dataset. BCL2L1 and BIRC2 were significantly downregulated, and the remaining 12 were significantly upregulated. The results of the GSE123568 dataset and the GSE74089 dataset were consistent. Finally, we validated 14 biomarkers by collecting clinical samples. The analysis revealed that five of these molecules exhibited significant statistical relevance, and their expression trends were consistent with our previous findings.

BCL2-xL (gene/transcript name BCL2L1) is a member of the BCL-2 family and functions as a prevalent anti-apoptotic protein, promoting cellular survival (Opferman and Kothari, 2018). A study demonstrated that, compared to wild-type, transgenic mice overexpressing BCL-XL exhibited an increase in both trabecular and cortical bone volume, and a concurrent reduction in osteoblast apoptosis (Moriishi et al., 2016).

AKT1, a crucial intracellular signaling molecule, encodes a serine/threonine kinase that plays a significant role in regulating the development, strength, and integrity of normal bones by phosphorylating various downstream target proteins (Cho et al., 2001; Mukherjee et al., 2014). Studies have reported that AKT1 not only aids the survival of cells exposed to apoptotic stress but also promotes angiogenesis by influencing various angiogenic factors (Somanath et al., 2006; Sun et al., 2020). The expression of AKT1 in NFH patients might indicate the body’s response to adverse environmental conditions by activating it, thereby facilitating the repair and regeneration of damaged tissues. Additionally, AKT1 plays a vital role in glucose metabolism by phosphorylating and activating key glycolytic enzymes, such as hexokinase and phosphofructokinase-1, thereby accelerating the glycolysis process (Lee et al., 2017). In a study on gastric cancer, cancer cells increased AKT1 expression to enhance their proliferation and glycolytic capacity (Pan et al., 2019). Correspondingly, under hypoxic conditions, as observed in NFH, osteoblasts and osteocytes may overly rely on glycolysis for energy production (Huffman et al., 2007). AKT1’s promotion of glycolysis aids in sustaining the cellular energy requirements in hypoxic environments.

CASP8, a member of the cysteine-aspartic acid protease (caspase) family, plays a pivotal role in apoptosis and immune system functions (Fritsch et al., 2019). It primarily initiates extrinsic apoptosis, whereupon the activation of CASP8 occurs following the binding of death receptors (such as Fas) to their ligands, subsequently activating other caspases (like CASP3), culminating in apoptosis (Orning and Lien, 2021). Moreover, CASP8 significantly influences the activation and maturation of T lymphocytes. Studies have shown that patients with inflammatory bowel disease characterized by hereditary Casp8 deficiency exhibit T lymphocyte dysfunction (Becker et al., 2013; Lehle et al., 2019). In bone metabolism, T lymphocytes regulate the balance between bone resorption and formation through the secretion of OPG and RANKL (David, 2007). In a retrospective clinical study, researchers observed a notable increase in lymphocyte counts and CD3T lymphocytes in NFH patients compared to healthy individuals (Ma et al., 2019), suggesting that alterations in CASP8 might reflect the immune system’s state, particularly T lymphocytes, in NFH. Furthermore, CASP8 also enhances inflammation by activating relevant cytokines (Phillips et al., 2016). Studies have confirmed that CASP8 directly participates in the cleavage and activation of IL-1β during the maturation of IL-1β in dendritic cells and macrophages under endoplasmic reticulum stress (Bossaller et al., 2012; Shenderov et al., 2014).

TNFRSF1A, a receptor for tumor necrosis factor alpha (TNFα), plays a pivotal role in regulating inflammatory responses and apoptosis through mediating TNF-α signal transduction (McDermott et al., 1999). In the context of bone homeostasis, TNFα′s interaction with TNFRSF1A contributes to the promotion of osteoclastogenesis and the suppression of osteoblast activity (Osta et al., 2014). Furthermore, research on peripheral nerve injury revealed that TNFRSF1A, BIRC2, and BIRC3 in Schwann cells (SC) assume an anti-apoptotic function by modulating NFκB signaling pathway in response to inflammatory stimuli, thereby mitigating peripheral nerve damage (Wang et al., 2012).

XIAP, BIRC2, and BIRC3, members of the inhibitor of apoptosis proteins (IAP) family, are crucial in apoptosis inhibition (Jost and Vucic, 2020). In spinal cord injury (SCI) research, it was observed that XIAP overexpression diminished neuronal caspase activity and apoptotic cell death (Reigada et al., 2023), suggesting that XIAP exerts anti-apoptotic effects by curtailing specific caspase functions (Shi, 2004). XIAP may thus have a protective and reparative role in NFH(Mollazadeh et al., 2015).

STAT3, belonging to the STAT protein family, is a key intracellular signaling molecule vital for inflammation, apoptosis, bone tissue repair and regeneration, and angiogenesis (Zhou et al., 2021). Investigations have shown that STAT3 hampers apoptosis by enhancing the expression of Bcl-2 family anti-apoptotic proteins (Caldera et al., 2008)​​. Moreover, it augments osteogenic differentiation of BMSCs and fosters BMSC-mediated angiogenesis (Chen et al., 2021). The heightened expression of STAT3 in NFH suggests its necessity in fostering new blood vessel formation to alleviate ischemia in necrotic regions and aid in repairing damaged areas.

Immune infiltration analysis indicated a higher proportion of Neutrophil cells and Macrophage M1 cells in the NFH group compared to normal individuals. A primary contributor to NFH is diminished blood flow to the femoral head, resulting in local cellular demise and subsequent inflammation (Margraf et al., 2022). Neutrophils are likely recruited to the affected site as part of the inflammatory response, facilitating the removal of necrotic cells and debris. Moreover, research suggests that Neutrophils can express RANKL and OPG, playing roles in both bone and vascular remodeling (Schruefer et al., 2005; Poubelle et al., 2007). This ability might account for their increased presence in NFH. Macrophage M1 cells, predominantly involved in inflammation (Yunna et al., 2020), become more prevalent in advancing stages of NFH. This shift results in heightened local inflammatory mediators (including IL-1β and TNF-α) and accelerates bone cell mortality (Cheng et al., 2023). Studies have demonstrated that human umbilical cord mesenchymal stem cells convert Macrophage M1 to Macrophage M2 via the AKT/mTOR signaling pathway, mitigating necrosis and osteocyte apoptosis in steroid-induced NFH models (Tian et al., 2022). Subsequent to our analysis, four central immune cells (Neutrophil, Mast cell resting, Myeloid dendritic cell activated, Macrophage M0) were identified utilizing LASSO and RFE algorithms. Correlation analysis between 14 key hub genes and these immune cells revealed a strong positive correlation between STAT3 and Neutrophil cells (r = 0.6804, p = 3.525e-05), and a marked negative correlation between XIAP and activated Myeloid dendritic cells (r = −0.3610, p = 0.04003). Prior research has established that STAT3 facilitates the differentiation of bone marrow progenitor cells into Neutrophil cells while inhibiting their transformation into mononuclear macrophages (Zhang et al., 2023). Given STAT3’s directive role and Neutrophil cells’ significant involvement in inflammatory responses (Margraf et al., 2022), this interaction may significantly influence NFH through mechanisms related to inflammation and immune cell dynamics. Activated Myeloid dendritic cells, pivotal components of the immune system, are crucial in modulating both immune and inflammatory responses (Chistiakov et al., 2015). In the context of NFH, they might exacerbate tissue damage by fostering an inflammatory response. XIAP, functioning as an anti-apoptotic protein that impedes apoptosis (Jost and Vucic, 2020), could safeguard bone cells from harm, potentially decelerating NFH progression. An upsurge in XIAP expression might imply reduced apoptosis, thereby preserving bone tissue integrity. Concurrently, this could diminish the activation necessity of bone marrow-derived dendritic cells, as decreased cellular damage might result in lower inflammation levels. The results of our study provide valuable clues for further study of the pathophysiological mechanism of NFH on immune infiltration.

The main limitation of this study lies in the failure to collect and analyze longitudinal data. Due to the high costs of long-term follow-up and the challenges in data completeness caused by individual patient differences, the implementation of the study was difficult. Additionally, the study design did not directly link biomarkers with clinical outcomes and disease severity. It primarily focused on the identification and preliminary characterization of biomarkers without conducting large-scale clinical etiological research. In the future, we will focus on improving the study design by conducting longitudinal data collection to explore the temporal dynamics of gene expression and immune responses in disease progression, strengthen the association analysis with clinical outcomes, and enhance subsequent clinical etiological research.

## Conclusion

In summary, this study utilized bioinformatics analysis to investigate immune cell types in NFH, identifying several hub genes. Experimental validation confirmed that CASP8, TNFRSF1A, AKT1, XIAP and STAT3 can serve as potential biomarkers for early diagnosis, understanding of pathogenic mechanisms, and therapeutic strategy development in NFH. Furthermore, our research also explored the associations between these hub genes and immune cells.

## Data Availability

The datasets presented in this study can be found in online repositories. The names of the repository/repositories and accession number(s) can be found in the article/Supplementary Material.
